# Cohesive Gold

**Published:** 1885-02

**Authors:** J. Smith Dodge


### COHESIVE GOLD.

BY J. SMITH DODGE, JR., M. D., D. D. S.

The phrase "cohesive gold" contains a history, and without the history the phrase would be superfluous. It is as much a property of gold to be cohesive as to be yellow, only the yellowness has been always known, while the cohesiveness is a late discovery. The quality, therefore, intrinsic though it is, may be easily concealed, either by alloy or by superficial uncleanness, so that the very first demand for cohesive gold is that it should be pure within and without. The inward purity must depend on the manufacturer, but the dentist can himself secure a clean surface, and both are absolutely indispensable. I forget exactly what small percentage of alloy gold is said to bear without losing its cohesion. But I hope the manufacturers are not looking into this, for we want gold "1,000 fine," and nothing else. It should be understood that it is really this, and not any secret of concoction, that makes one preparation better than another. Some sort of ingenious jugglery may make a slightly alloyed gold cohere, but given a good honest article, as pure as chemistry can make it, and when the surface is clean, it *must* stick.

Nor do I believe there is as much in the form of preparation as is often supposed. It is certain that somebody has been able to make superior fillings with each kind of gold that has ever been popular; and it is equally certain that nobody has used them all with the same success. It appears, therefore, that the value of each depends rather on its finding a hand to which it is just suited, than on any intrinsic superiority of the gold, provided always it be pure. Still, there is a plain reason for the very general preference given to foil over all other forms of cohesive gold. There is so much less surface to a given weight of gold that the labor of bringing all surfaces into contact is much less. Besides, it is no small part of success in filling to put just the right quantity of gold at a time under the plugger, and this is more easily and certainly done with foil than with anything else.

The question again of thick foil or thin is very much a matter of personal adaptation, but it is certain that anything can be done with thin foil which is possible with thick, while for many uses of thin foil thick foil is out of the question. Probably if a man were carefully forming his habits so as to get the best results with the least labor, he had better accustom himself to use thin foil for accuracy, and thick for speed.

The manufacturer having furnished the proper gold, the dentist should purify its surface. To be sure, one can buy cohesive foils ready for use, and many dentists are satisfied with them. But exposure to the air is sure to deposit moisture or whatever else on the surface, and a sufficient length of exposure will render any gold non-cohesive. Therefore, there is only one period at which the gold is equally cohesive, time after time, and that is just after it has been purified. But if my gold is less cohesive to-day than it was yesterday, it needs more force to make a solid filling, and so I must either vary my habits of working or send away fillings of different degrees of excellence. For these reasons I prefer to make ready my gold at no great time before it is used, and do not ask the manufacturer whether he is pleased to call it cohesive, or semi-cohesive, or non-cohesive. Let him make it pure and I will make it stick. It is a pity that this has come to be called annealing. It resembles that process to be sure, in its method, but the object is simply to cleanse the surface and not to make a molecular change in the metal. It is very usual to anneal the gold by passing each pellet or ribbon through the flame on its way to the cavity, but I believe this a faulty method. It cannot possibly secure a uniform degree of heat; it may easily melt thin edges into lumps, and it spoils the temper of the instruments used. My plan, which I have followed with success for over twenty years, is to anneal all my gold at once, immediately, or not long, before using, on a pan of thin platinum, which is made as follows: a piece of platinum two and a half inches square and in thickness No. 34, is turned up an eighth of an inch all round. A platinum wire, No. 15, is split at one end like Y and the angle soldered with gold to one corner of the pan, after which the wire is set tightly into a wooden handle, and the pan is complete. It is necessary to have as little thickness as possible at all parts of the pan, that it may heat easily. The gold (in pellets) is put into the pan and heated over a flame, and the right degree of heat is reached when the platinum becomes dull red. Of course it is not necessary to redden the whole pan at once, but all on which any gold lies should be in turn brought to this color. The gold is then turned out upon a plate of glass, and is so cohesive that a single pellet held in the forceps will pick up all the rest, like filings hanging from a magnet. Of course the pellets have to be separated before using, which is a slight task, and when it is done I can begin with gold enough before me to make a two hours' filling, all in the highest state of cohesiveness.

The pellets which I use are Williams' cylinders, ranging from No. $\frac{1}{4}$ to No. 2; but I attach no importance to the source of my gold, except that it be honestly pure and always the same, nor to the form except that it be convenient.

With such gold so prepared, the practiced hand can make fillings which, in their proper place, far excel all other dental restorations.

